# PLA-Based Materials Containing Bio-Plasticizers and Chitosan Modified with Rosehip Seed Oil for Ecological Packaging

**DOI:** 10.3390/polym13101610

**Published:** 2021-05-17

**Authors:** Raluca Nicoleta Darie-Niță, Maria Râpă, Morten Sivertsvik, Jan Thomas Rosnes, Elisabeta Elena Popa, Raluca Petronela Dumitriu, Octaviana Marincaș, Ecaterina Matei, Cristian Predescu, Cornelia Vasile

**Affiliations:** 1 “Petru Poni” Institute of Macromolecular Chemistry, Physical Chemistry of Polymers Department, 41A Gr. Ghica Voda Alley, 700487 Iasi, Romania; darier@icmpp.ro (R.N.D.-N.); rdumi@icmpp.ro (R.P.D.); cvasile@icmpp.ro (C.V.); 2 Faculty of Materials Science and Engineering, University POLITEHNICA of Bucharest, 313 Splaiul Independentei, 060042 Bucharest, Romania; ecaterinamatei@gmail.com (E.M.); cpredescu56@yahoo.com (C.P.); 3 Nofima AS, Department Processing Technology, Richard Johnsens gt 4, 4068 Stavanger, Norway; Morten.Sivertsvik@nofima.no (M.S.); thomas.rosnes@nofima.no (J.T.R.); 4 Faculty of Biotechnology, University of Agronomic Sciences and Veterinary Medicine Bucharest, 59 Marasesti Blv., District 1, 011464 Bucharest, Romania; elenush_68@yahoo.com; 5 The Faculty of Orthodox Theology “Justinian the Patriarch”, University of Bucharest, Sf. Ecaterina Street, no. 2, Sector 4, 040155 Bucharest, Romania; octavianamarincas2019@gmail.com

**Keywords:** polylactic acid, bio-plasticizers, chitosan, rosehip seed oil, biocomposites, antibacterial, antioxidant, eco-friendly packaging

## Abstract

Several recipes based on PLA, bio-plasticizers, and active agents such as vitamin E and cold-pressed rosehip seed oil encapsulated into chitosan by the emulsion method named here as chitosan modified (CS-M) were elaborated by melt compounding for food packaging applications. Resulted biocomposites have been investigated from the point of view of physical-mechanical, thermal, barrier, antimicrobial, and antioxidant properties to select the formulations with the optimum features to produce food trays and films for packaging applications. The obtained results showed that the elaborated formulations exhibit tensile strength and flexibility dependent on their composition being either rigid or flexible, as well as antimicrobial and antioxidant activity, which will potentially lead to prolonged use for food packaging. The recipe with PLA matrix and 40:60 Lapol^®^108 as masterbarch/polyethylene glycol (MB/PEG) bio-plasticizers ratio was distinguished by an improvement of over 100 times in terms of flexibility compared with neat PLA, while the highest antioxidant activity (36.27%) was recorded for the sample containing a CS-M and MB/PEG ratio of 60:40. An enhancement of ~50% for the water vapor barrier was recorded for PLA/CS-M_100:0 material. By modulating the MB and PEG bio-plasticizers ratio, the design of new eco-friendly food packaging materials with antimicrobial/antioxidant characteristics by using the existing technologies for processing synthetic polymers (melt mixing, compounding, pressing, thermoforming) has been successfully realized.

## 1. Introduction

With the steady growth of the global population, the food packaging market is constantly evolving to meet safety and environmentally requirements. Food quality could also be ensured by specific packaging materials that simultaneously require adequate mechanical, thermal, optical, barrier and antibacterial properties [1]. The market is mainly based on food packaging made on conventional polymers such as polyethylene (PE), polystyrene (PS), polypropylene (PP), and polyethylene terephthalate (PET) processed by extrusion, thermoforming, or blown casting technologies. Although these traditional plastics have physical and mechanical properties suitable for food packaging, they are not biodegradable, and are usually obtained from limited fossil resources. Consequently, the adoption of biodegradable materials from renewable resources for food packaging that can be processed by existing technologies of conventional polymers will lead to substantial benefits for the environment in terms of overall carbon footprint and reduction of waste.

One of the best solutions to reduce food waste is to increase the shelf-life of the packaged food without compromising food safety. Currently, research trend focuses on active materials for packaging of fresh food (such as meat, fish, cheese, fruit or vegetables), based on biodegradable polymer materials containing additives with antimicrobial and/or antioxidant properties that could inhibit microbial contamination of food products [2]. A variety of synthetic or natural antimicrobial agents can be incorporated into packaging materials [3], among which one can mention: nisin, chitosan, herbal essential oils, rosemary (*Rosmarinus officinalis*), oregano (*Origanum vulgare*), sage (*Salvia officinalis*), rosehip [4], and medicinal and aromatic plants [5]. The antimicrobial activity of these agents is attributed to some classes of compounds which constitute volatile fraction, such as hydrocarbons terpenes, oxygenated terpene, aldehydes, ketones and esters, carnosic acid, carnosol, rosmarinic acid, or others, like eugenol, carvacrol, and thymol. Several technologies can be adapted for utilization of antimicrobial agents, namely: either directly into an active sachet placed within the package, or through incorporation into the composition of the packing material during processing, by coating of the packaging surface, or by using antimicrobials polymers which exhibit film-forming properties [6,7]. The active substance incorporated into packaging by any technique, in contact with food, should be released, controlling the specific rate for a given packaged food product to inhibit microbiological or oxidative degradation.

There is an increased awareness among consumers on environmental protection and thus, the demand for biodegradable food packaging made from recycled materials or renewable resources. Among the bio-derived materials used in packaging applications, polylactic acid (PLA) has received special attention in recent decades as one of the most attractive short-use packing materials due to its biodegradability, good transparency, high modulus and rigidity at room temperature and lack of ecotoxicity [8]. PLA requires small amount of energy for its production compared to oil-based polymers, being a biopolymer chemically produced from renewable resources, especially from sugar and starch or cellulose. PLA presents barrier properties comparable to those of PET. PLA is potentially degradable in the soil, compost or in the human body by hydrolysis. In the degradative process, after 40–60 days at 50–60 °C, PLA is hydrolyzed into small molecules (oligomers, dimers, monomers) which are further degraded in CO_2_ and H_2_O by microorganisms from the compost or soil. Packaging materials based on PLA are therefore considered safe materials both in terms of food contact, as well as for environmental protection [9]. PLA can be used to produce semi-rigid thermoformed trays, films, and bottles, but due to its thermal and barrier properties it cannot be employed for packaging hot or carbonated liquids. Beside beneficial features, PLA exhibits however limitations for its use in the food packaging due to the low water vapor and oxygen permeability, inferior mechanical and thermal properties, fragility and heat deflection temperature. PLA’s drawbacks can be exceeded by improving its properties through its modification with plasticizers like poly (ethylene glycol) (PEG), citrate esters, glycerol triacetate, monoesters of glucose, fatty acid esters, lactide and oligomers of lactide, vegetable oils etc. [10,11,12,13,14,15], blending with other polymers, such as poly (butylene adipate-co-terephthalate) PBAT [16], poly(hydroxybutyrate) [17], elastomers [18], copolymerization and incorporation of the fillers [19,20,21]. Selection of plasticizers for modifying the properties of biopolymers used in food packaging is limited by the technical and legislative requirements (Directive 2002-72-EC) relating to plastic materials and articles intended to come into contact with food products. Use of plasticizers leads to significant improvement in elongation, to the detriment of tensile strength, but also to possible migration in time out of PLA matrix. Multifunctional PLA materials containing PEG as plasticizer and powdered rosemary ethanolic extract (up to 0.75 wt.%) as natural have been investigated by Darie-Nita et al. [22]. It was reported that the increasing of rosemary ethanolic extract in PLA-based materials has a positive effect on the migration of active components, barrier properties, as well as antioxidant and antimicrobial properties.

Chitosan (CS) represents another biopolymer which can be used for packaging purposes due to its antimicrobial activity, non-toxicity, and biodegradability. It is the second most widespread polysaccharide in nature after cellulose, being obtained by alkaline deacetylation of chitin. Chitosan is widely used for obtaining of antimicrobial film for edible coatings, reducing water vapor and oxygen permeability, diminishing the rate of breathing and increasing the shelf life of the fruits [23]. Several studies reported coatings of PET with chitosan as a potential active packaging material for protecting meat against *Salmonella enterica*, *Campylobacter* spp., *Listeria monocytogenes*, *Escherichia coli*, and *Candida albicans* [24] or materials with chitosan incorporated in low density polyethylene (LDPE) [25,26], which increases the shelf life of poultry meat. Antimicrobial nature of chitosan is mainly due to its positively charged amino groups that interact with negatively charged microbial cell membranes [27]. In the case of PLA/chitosan formulations, some might present drawbacks regarding the dispersion of chitosan in the PLA matrix, the thickness and appearance of the film, the tendency of the plasticizer to migrate over time or to be extracted by food simulants (acetic acid) as well as the decrease in the tensile strength and elongation at break [28]. These limitations occur due to the fact that chitosan is a naturally hydrophilic polymer, while PLA is hydrophobic. The elastic properties of chitosan could be improved by its plasticization with PEG [29], some resulting materials being stable until nine weeks of storage, as observed by Caner et al. [30]. Butnaru et al. [31] reported the preparation of films based on chitosan and cold-pressed rosehip seed oil by the encapsulation emulsion method. Accordingly, the incorporation of cold-pressed rosehip seed oil in chitosan resulted in flexible films with improved mechanical, gas, and water vapor barrier properties, as well as antioxidant and antibacterial activity against *Escherichia coli*, *Salmonella typhymurium*, and *Bacillus cereus*.

Vitamin E has been used as antioxidant in order to increase the shelf life of packaged foods and consequently reduce the use of preservatives in food [25,32].

The literature has noted several approaches related to the incorporation of CS into PLA for potential packaging materials. Thus, Bonilla et al. [33] observed that the addition of 5% or 10% chitosan did not affect the thermal properties of PLA, but decreased film flexibility, while Spiridon et al. [34] realized green materials by melt processing with relatively good mechanical and thermal-mechanical properties by incorporating keratin in PLA/CS blends, followed by changes evaluation after accelerating weathering conditions. Extruded films of PLA with 0.5–2% CS were found promising for preserving refrigerated fish [35]. Introduction of chitosan into plasticized PLA with tributyl o-acetyl citrate (ATBC) conducted to antimicrobial materials with tensile strength at break of ~24 MPa, and water vapor transmission rate of 43.91 g m^−2^ day^−1^ properties [36]. In other paper, Kasirajan et al. [37] reported the production of PLA/chitosan membrane with tensile strength of 17.809 MPa, elongation at break of 300.11%, and oxygen transmission rate of 1614.21 cm^3^/(m^2^·day·atm) from solvent casting method. Two-layer composite film containing rosin modified cellulose nanofiber in PLA coated with chitosan showed good mechanical properties and antimicrobial performance against *E. coli* and *B. subtilis* [38]. Recently, Li et al. [39] developed a three-layer composite film of PLA/SiOx/CS characterized by a water vapor transmission rate of 111.6 g m^−2^ day^−1^ and enhanced antibacterial activity against *E. coli* and *S. aureus*.

The present paper reveals the producing of several new recipes based on PLA, Lapol^®^108 as masterbarch/polyethylene glycol (MB/PEG) bio-plasticizers and active agents as vitamin E and cold-pressed rosehip seed oil encapsulated into chitosan by melt mixing technology. By modulating the MB and PEG bio-plasticizers ratio, materials with improved flexibility/rigidity, as well as antioxidant, antimicrobial, and barrier properties as required for food packaging are envisaged to be obtained. To the best of authors’s knowledge, such approach of combining these materials with synergetic properties has not been reported before.

Compared to already existing materials, an innovative aspect of the present study consists in the use of rosehip seed oil encapsulated into chitosan as a double bioactive agent in the development of biocomposites for food packaging. It is expected that the addition of rosehip seed oil, which acts also as antioxidant, antibacterial and plasticizer for chitosan, will be responsible for improving the processability of PLA-based materials. The interfacial compatibility between chitosan and the thermoplastic matrix is an essential requirement for obtaining favorable mechanical properties. Therefore, another innovative feature is related to the improvement of the immiscible PLA-chitosan interface by the use of two commercial bio-plasticizers, a masterbatch obtained by the compounding of PLA with Lapol^®^108 bio-plasticizer (which is a polyester plasticizer), having both the plasticizer and compatibilizer roles [15] and PEG in different ratios.

The aim of this paper is thus to obtain PLA-based biocomposites containing bio-plasticizers and bioactive agents, such as rosehip seed oil encapsulated into chitosan and vitamin E with physical-mechanical, thermal, barrier, antimicrobial, and antioxidant properties appropriate to use for food packaging by melt mixing technology on similar equipment to that used for processing conventional polymers. The selection the best formulations for designing and producing food trays or flexible film for food packaging applications is also addressed.

## 2. Materials and Methods

### 2.1. Materials

To make the active biocomposites, the following raw materials were used:

(i) Polylactic acid (PLA) Ingeo^®^ Biopolymer 2003D (NatureWorks LLC, Minnetonka, MN, USA), pellets, biopolymer obtained from renewable resources, with a 4% content of D-lactide, density of 1.24 g cm^−3^ and a melt flow index (MFI) of 5–7 g/10 min (at 210 °C/2.16 kg). The Food and Drug Administration (FDA) approved its utilization in food packaging applications.

(ii) Medium molecular weight chitosan (Sigma Aldrich, Darmstadt, Germany) was used to encapsulate cold-pressed rosehip seed oil (CS-M) by the emulsion method [31]. Shortly, this method has two steps. First, chitosan/rosehip seed oil emulsion was obtained by mixing 2 mL of rosehip seed oil with 0.375 g polyoxyethylene sorbitan monooleate (Tween 80) and 85 mL chitosan solution (3%, *w*/*v*) (chitosan dispersed in acetic acid solution (5%, *v*/*v*)) and homogenization by ultrasonic treatment. Then, films with smooth surfaces and uniform thickness of 0.148–0.193 mm were produced by casting emulsion in glass Petri dishes and subsequently drying. The used chitosan showed a deacetylation degree of 75–85%, viscosity of 200–800 cP and M_W_ = 190,000–300,000 g/mol.

(iii) Masterbatch concentrate in PLA (MB) (LAPOL, LLC, Santa Barbara, CA, USA) supplied as a pre-compounded masterbatch, between 30% Lapol^®^108 resin and 70% PLA [40]. It is generally used to improve the melt processability by extruding and thermoforming of polymeric recipes due to the compatibility and miscibility with PLA.

(iv) Polyethylene glycol BioULTRA 4000 (PEG) (Sigma-Aldrich, Saint Louis, MO, USA), in the form of pellets, has a molecular weight (calculated from the OH index) of 4016 g/mol and a melting point of 61 °C. PEG is a specialty polymer approved as biodegradable [41]. PEG biodegradability depends on its molecular weight and types of used microorganisms. Full biodegradability of PEG of up to 13,000–14,000 molecular weights in aerobic pure culture of a strain of *Pseudomonas stutzeri*, a river water bacterial isolate, has been reported [42].

(v) Vitamin E (±α-Tocopherol), a bioactive agent (Sigma-Aldrich, Saint Louis, MO, USA), had a density of 0.95 g cm^−3^ (20 °C).

### 2.2. Production of Materials

The experimental design of our study was based on initial processing of several new formulations containing ecological components on the Brabender Plastograph, followed by their complex characterization for selection of two final recipes with the best overall performance for developing at laboratory level of a food packaging tray and a film. The second production step consisted of obtaining pellets by compounding at a larger scale (200 kg) from the selected two recipes to be further used for trials to obtain the tray by thermoforming, as well as covering film (by pressing) for the developed trays.

#### 2.2.1. PLA Biocomposites Processing

The processing of the polymer blends consisted in bringing the raw materials in molten state in a Brabender Plastograph, provided with a mixing chamber of 50 cm^3^, at a temperature of 170 ± 5 °C, mixing for 6 min in order to avoid the thermal degradation of components, with screws speed of 60 rotations per minute. Counter rotating rotors were used for processing. PLA is very hygroscopic and might retain moisture from the air, leading to degradation of macromolecular chains, reducing product viscosity and resistance. Therefore, prior to use, the PLA and MB were dried for 24 h in an oven with air circulation at 50 °C (moisture content <200 ppm).

Chitosan encapsulated with cold-pressed rosehip seed oil has been dried at 40 °C for 4 h in an oven with air circulation. The homogenized melted mixtures were hot-pressed on a laboratory press under the following conditions: preheating for 5 min at 100 bar and pressing for 10 min, at a pressure of 147 bar, both at 175 °C, followed by cooling for 20 min and 147 bar in order to obtain thin homogeneous films with dimensions of 200 × 200 × 0.1 mm and homogeneous plates with dimensions of 150 × 150 × 1 mm. Specimens were prepared from these plates for testing of tensile, differential scanning calorimetry (DSC) and antioxidant properties, and films for Fourier transform-infrared spectroscopy (FT-IR), UV/VIS spectroscopy, water vapor transmission rate (WVTR), antioxidant activity, and antimicrobial features.

The compositions of the resulted materials based on PLA, plasticizers, cold-pressed rosehip seed oil encapsulated into chitosan named chitosan modified (CS-M) and vitamin E are presented in Table 1.

All materials have been designed with different ratios between MB and PEG (100:0, 70:30, 60:40 and 40:60) as the ratio between PLA and bio-plasticizers (MB or MB+PEG) being of 80/20 wt%. The amount of used CS-M was 1 wt%.

The samples prepared by melt blending on the Brabender Plastograph were further investigated in order to select the most suitable formulations for a packaging tray and film. For mechanical and thermal characterizations, neat PLA, PLA modified with MB and unmodified chitosan and vitamin E, respectively (PLA, PLA_100:0, and PLA/CS_100:0) were included for comparative purposes.

#### 2.2.2. Development of Food Packaging System: Compounding and Thermoforming

The designed food packaging system contains a food tray and film obtained by compounding of the two optimal formulations (PLA/CS-M_60:40 for tray and PLA_40:60. For film) selected based on complex characterization of the blends processed in Brabender.

**Scheme 1:**
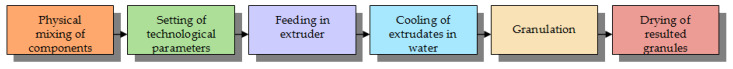
Technological phases of the compounding process.

Several steps have been approached for achieving the final food packaging materials, namely:

(i) Compounding at larger scale. A twin-screw extruder, type TSE 35 (diameter screw = 35.6 mm, L/D = 32–48, with granulation line) was used for compounding of two selected biocomposites. The technological phases in the process of compounding the components consisted in: obtaining a physical mixture between components, setting technological parameters (screw speed, temperature per zones, pressure), feeding the mixture into the extruder, cooling the extruded wires in the water bath, and granulating or cutting the wires (speed = 600 rpm) according to Scheme 1. Pellets of PLA/CS-M_60:40 and PLA_40:60 resulted in this process.

(ii) Obtaining of sheets and films. Sheets and films with 30 × 30 × 0.05 cm and 30 × 30 × 0.01 cm dimensions respectively were obtained from the PLA/CS-M_60:40 and PLA_40:60 compounds obtained at larger scale by using a laboratory press at a pressure of 147 bar and temperature of 175 °C.

(iii) Thermoforming of food trays. For this step a prototype matrix with two cavities was designed with CAD software having dimensions of 140 × 70 × 40 mm. Previously PLA/CS-M_60:40 sheets with the dimension of 30 × 30 × 0.05 cm were used for obtaining of food trays.

### 2.3. Investigation Methods

#### 2.3.1. Thermogravimetric Analysis

Thermogravimetric analysis for cold-pressed rosehip seed oil and vitamin E was realized by means of a STA 449 F1 Jupiter apparatus (Netzsch STA 44F1, Germany) with a heating program from 30 °C up to 600 °C, at a 10 °C min^−1^ heating rate, under nitrogen as a purge and protective gas (flow rate of 40 mL min^−1^). The temperature reproducibility of TG was ±2 °C, and the non-volatile fraction was ±3%. A sample of ~15 mg was placed in Al_2_O_3_ crucible for each recording. Temperature calibration was done using 99.99% purity standard indium, zinc, tin, bismuth, and aluminum.

#### 2.3.2. Melt Processability

The melt processing behavior of the developed PLA-based materials was evaluated by analyzing the processing characteristics from the torque-time curves registered during mixing on the Brabender mixer by means of maxim torque (TQ_max_), final torque (TQ_end_), melt viscosity (η) and power consumption (P) [43]. The melt viscosity (η) can be estimated as the ratio of torque to rotor speed and the power consumption (P) is given by the multiplication of torque with rotor speed, according with Equations (1) and (2) [44]:

η = K(TQ_end_/S) (N m rpm^−1^) (1)

P = TQ_end_ × 2πS/60 (W) (2)

where K is a constant depending on temperature, TQ_end_ is torque (N m), and S is the rotor speed (rot/min).

#### 2.3.3. Fourier Transform Infrared Spectroscopy (FT-IR)

The film samples obtained by processing with a Brabender Plastograph and hot-pressed were analyzed by a FTLA 2000-104 spectrophotometer (ABB, Bomem Inc., Quebec, QC, Canada) device in attenuated total reflectance mode (ATR-FT-IR) using a ZnSe crystal with an incidence angle of 45°. All spectra represent the average of 20 scans recorded at 4 cm^−1^ resolution in a range from 4000 to 750 cm^−1^, using air as background. Prior measurements, the materials were conditioned at 60% ± 2% relative humidity and 23 ± 2 °C for 48 h.

#### 2.3.4. UV/VIS Spectroscopy

The transmittance of neat PLA, PLA:60:40, PLA/CS-M:60:40, and PLA_40:60 films processed by hot-pressing of mixtures obtained by Brabender plastograph was assessed by ultraviolet-visible (UV-VIS) spectroscopy (Orion AquaMate 8000, Thermo Scientific Water Analysis Instrumentation, Chelmsford, MA, USA) in the 300–800 nm wavelength.

#### 2.3.5. Stress-Strain Measurements

The tensile properties (tensile strength, elongation at break, the Young’s modulus) were determined according to EN ISO 527-2:2011 using an Instron 3345 tester (Norwood, MA, USA) on specimens with thickness of 1 mm and length of 40 mm taken from plates obtained by hot-pressing of Brabender formulations. Test machine operated at a crosshead speed of 10 mm/min. At least five specimens were tested for each composition and the average value is reported.

#### 2.3.6. Differential Scanning Calorimetry (DSC) Measurements

Thermal analysis of samples taken from plates obtained by hot-pressing of Brabender formulations was carried out on a DSC analyzer (823^e^ (Mettler Toledo, Greifensee, Switzerland) in the range of 35–190 °C and at a heating rate of 10 °C/min. From DSC curves, the second heating run, the glass transition temperature (Tg), cold crystallization temperature (Tcc), cold crystallization enthalpy (∆Hcc), melting temperature (Tm) and melting enthalpy (∆Hm) were determined. The degree of crystallinity (Xc) was calculated according to Equation (3), where the enthalpy value for a theoretically 100% crystalline PLA (ΔHm°) is 93.1 J/g [45]). The weight fraction of PLA from each sample was used for Xc calculation.

(3) $$\mathrm{Xc}\;(\%)=(\Delta H_{m}-\Delta H_{\mathrm{cc}})/\Delta {H_{m}}^{{}^{\circ}}\times 100$$

#### 2.3.7. Water Vapor Transmission Rate (WVTR)

Water vapor transmission rate (WVTR) of the film samples was determined with PBI-Dansensor L 80-5000 (Næstved, Denmark), at a temperature of 23 °C, 85% humidity (RH) and atmospheric pressure. The specimens with dimensions (108 × 108 × 0.1) mm taken from films obtained by pressing of Brabender formulations were used for testing of permeability. The results are presented as an average of five consecutive readings of the same value (±0.00).

#### 2.3.8. Antioxidant Activity Evaluation

The radical scavenging activity (RSA) of PLA-based composites films obtained by pressing of Brabender formulations was measured by 2,2-diphenyl-1-picrylhydrazyl (DPPH˙) radical scavenging assay. The DPPH test was performed on samples of ~250 mg for each composition tested, which were kept previously in vials containing 10 mL of methanol each, under continuous shaking (150 rpm) for 24 h at room temperature. From the methanolic solution obtained from each sample aliquots of 0.2 mL were extracted and mixed with 2 mL of DPPH solution (2 × 10^−4^ mol L^−1^). A mixture of 0.2 mL methanol with 2 mL DPPH solution was used as control. The mixtures were incubated in the dark at room temperature for 30 min before measurement. The radical scavenging activity of the samples was estimated by measuring the absorbance at 515 nm, using a Cary 60-UV–Vis Spectrophotometer (Agilent, Santa Clara, CA, USA). The antioxidant activity of the PLA-based composites was assessed based on the inhibition of DPPH radicals and calculated using the following equation:

(4) $$\%RSA=\left(1-\frac{A_{sample}}{A_{control}}\right)\times 100$$

where *A_control_* is the absorbance of the blank methanolic solution without sample and *A_sample_* is the absorbance measured for each sample tested.

#### 2.3.9. Antimicrobial Activity

Antimicrobial activity was performed in accordance with the ISO 22196:2007. The method involves placing a droplet of a suspension of either *Escherichia coli* ATCC 8739 or *Staphylococcus aureus* ATCC 6538 directly onto the films surface (50 × 50 mm) obtained by pressing of Brabender formulations. Each test specimen was prepared in a separate sterile Petri dish with the test surface uppermost and 0.4 mL of the test inoculum with concentration between 2.5 × 10^5^ cells/mL and 10 × 10^5^ cells/mL was pipetted onto the test surface. The test inoculum was covered with a piece of neutral film (without anti-bacterial properties, 40 × 40 mm) and gently pressed down on the film so that the test inoculum spreads to the edges. Immediately after inoculation, half of the untreated test specimens were processed by adding 10 mL of SCDLP (soybean casein digest broth with lecithin and polyoxyethylene sorbitan monooleate) broth to the Petri dish containing the test specimen and number of viable bacterial cells counted on Plate count agar. This value (U_0_) was used to determine the recovery rate of the bacteria from the test specimens. After the specimens were inoculated and the cover film applied, the lid of the Petri dish was replaced. After 24-h incubation at 35 °C and >90% relative humidity, the bacterial suspension was released from between the coverslip-test sample sandwich and the number of viable bacterial cells that had survived was determined for treated (At) and untreated specimen (Ut). When the conditions for a valid test were satisfied, the test has been deemed valid and the antibacterial activity was calculated using Equation (5).

R = (U_t_ − U_0_) − (A_t_ − U_0_) = U_t_ − A_t_ (5)

where R is the antibacterial activity; U_0_ is the average of the common logarithm of the number of viable bacteria, in cells/cm^2^, recovered from the untreated test specimens immediately after inoculation; U_t_ is the average of the common logarithm of the number of viable bacteria, in cells/cm^2^, recovered from the untreated test specimens after 24 h; A_t_ is the average of the common logarithm of the number of viable bacteria, in cells/cm^2^, recovered from the treated test specimens after 24 h.

#### 2.3.10. Statistical Analysis

Tensile properties and WVTR tests were evaluated by analysis of variance (ANOVA) (95% significant level) to find means that are significantly different from each other. The threshold significance level was set at 0.05. Thus, *p* (probability) values lower than 0.05 were considered to be statistically significant. Antimicrobial activity had a built-in validation test and only the validated results of three parallels were used. For tensile tests, WVTR and RSA, statistical analysis was performed by means of OriginPro 8 (OriginLab Corporation, Northampton, MA, USA). All data are presented as mean value with their standard deviation indicated (mean ± SD).

## 3. Results and Discussion

### 3.1. Thermogravimetric Analysis

Melt compounding of PLA with vitamin E (α-tocopherol) is a classic method for realizing materials with antioxidant properties [46,47].

**Figure 1:**
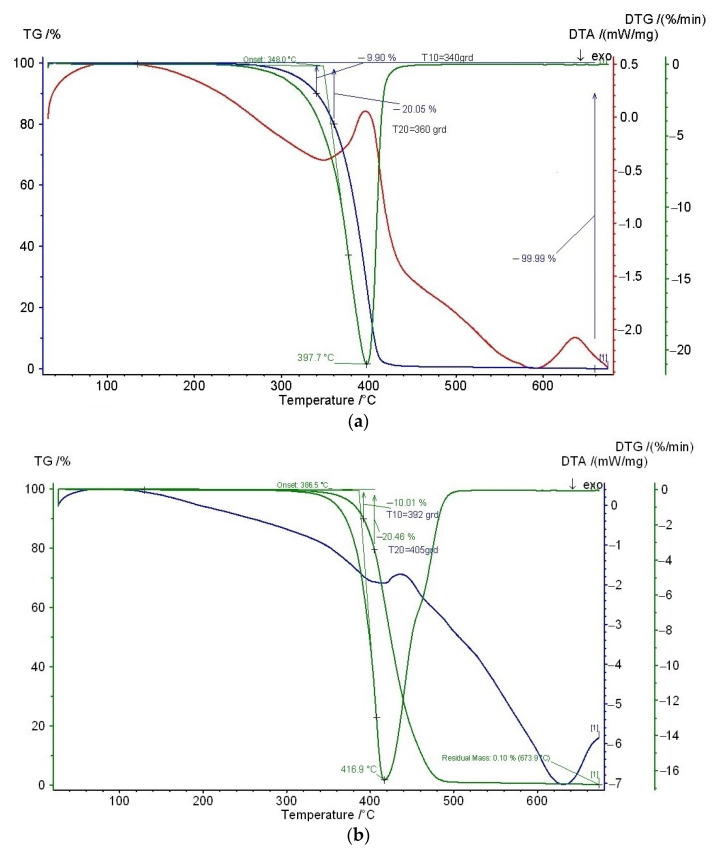
Thermal stability of (**a**) vitamin E, and (**b**) cold-pressed rosehip seed oil by means of thermogravimetric analysis.

Vitamin E and cold-pressed rosehip seed oil showed an initiation of thermal decomposition at 340 °C and 392 °C respectively (Figure 1), proving thermal stability during the Brabender processing at 170 °C. Early studies showed decomposition at 350 °C for α-tocopherol [48]. TGA results demonstrated that α-tocopherol improved the thermal stability of PLA films, thus allowing a better processability for PLA, which is beneficial for industrial processing. Thermal decomposition temperatures of 291 °C at 5% of weight loss and of 341 °C at maximum weight loss rate were registered for α-tocopherol [49]. In a previous study, we evaluated by thermogravimetry the thermal stability of PLA containing 5 wt% vitamin E and soybean oil the onset degradation temperature of 311 °C being recorded [50]. Melt stabilizing of α-Tocopherol has been proven, even at higher temperatures, while processing polypropylene at 260 °C and screw speed of 100 rpm [51].

Rosehip is a valuable source of bioactive compounds such as ascorbic acid, carotenoids, and phenolic compounds, being highly utilized in cosmetic, medical, pharmacological and food applications. Rosehip seed oil is abundant in polyunsaturated fatty acids (linoleic, oleic, and linolenic acids) and phytosterols [52], presenting a higher antioxidant capacity compared to the vegetable oils [53]. Contri et al. showed that the nanoencapsulation of rosehip in chitosan protected the oil from oxidation, resulting in a high stability formulation [54].

### 3.2. Processing Behavior

**Figure 2:**
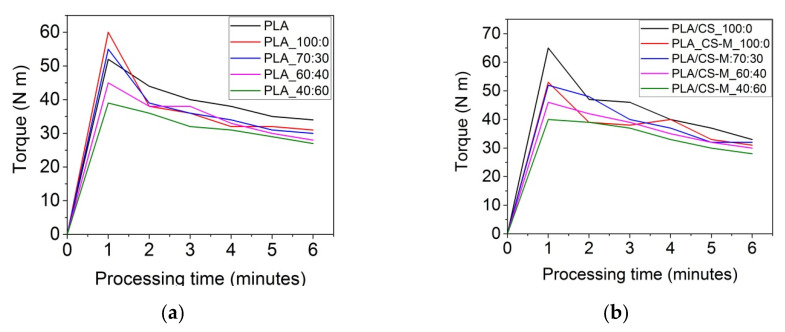
Torque-time curves for PLA-based materials processed by means of Brabender plastograph at temperature of 170 ± 5 °C and 60 rpm for 6 min for PLA with MB and PEG bio-plasticizers (**a**), and PLA-based plasticized composites containing MB and PEG bio-plasticizers in different ratios, CS-M and vitamin E (**b**).

The flow properties of polymeric blends for targeted applications can be adapted by controlling the rheological response of specific mixtures. Figure 2 displays the torque-time curves for all samples prepared by means of Brabender plastograph. The melt processability of PLA-based materials loaded with bio-plasticizers and simple chitosan or CS-M has been investigated by evaluating the maxim torque (TQ_max_), torque at the end of processing (TQ_end_), melt viscosity (η), and power (P) measured in the Brabender torque-time curves during blending (Table 2).

As observed from the data plotted in Table 2, the melt processing characteristics were influenced by the type of additives and their concentrations. PLA registered high values of torque and melt viscosity, respectively, requiring a high energy of mixing, due to constrained movements of the polymer chains imposed by PLA’s semi-crystalline structure. The introduction of unmodified chitosan and vitamin E to PLA/MB system (PLA/CS_100:0 sample) led to the increase of torque (33 N m) as compared with the sample without chitosan (31 N m) [15].

Very interesting was the behavior of CS-M on the PLA processability, which reduced the melt viscosity (0.51 N m/rpm), similar with that of PLA_100:0 sample. The increase of PEG content (diminishing MB) in the PLA/bio-plasticizers systems increased the PLA chain mobility, resulting in a profound effect on the melt stability by improving the blends melt flow. The presence of MB/PEG system in PLA matrix induced easy deformation of PLA chains in melt state, therefore a diminished frictional resistance [36]. As observed from the results plotted in Figure 2 and Table 2, the TQ_max_ was registered for all samples up to 1 min of processing, due to the initially solid state of PLA, MB, and PEG pellets. After that, the torque decreased as a consequence of their melting. The incorporation of 1% modified chitosan (CS-M) did not strongly affect the processing behavior of the respective blends, with only a slight increase of TQ_end_ and melt viscosity being registered. The samples containing the highest amount of PEG bio-plasticizer showed the best melt flow at the end of processing. Rosehip seed oil encapsulated into chitosan led to a reduction of the torque of respective formulations, for example 28 N m for PLA/CS-M_40:60 compared to 33 N m for PLA/CS_100:0 biocomposite. This can be explained by the composition of rosehip seed oil which acted as a multifunctional agent with the effect of improving processability, as well as antioxidant and antimicrobial properties. Moreover, following the behavior of samples during melt processing in Brabender Plastograph, it was observed that those containing CS-M did not stick on the device’s screws compared to the references. Improving the flow behavior improvement of the developed plasticized PLA/CS-M composites is very promising and important for the easy processing of materials intended for food packaging through various technologies such as plastic molding, extrusion, film blowing, etc.

### 3.3. FT-IR Results

**Figure 3:**
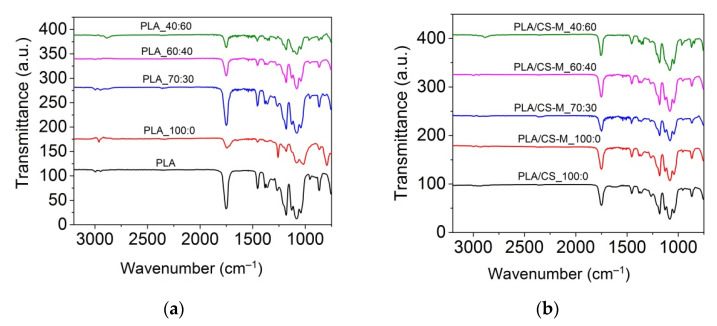
ATR-FT-IR spectra for PLA with MB and PEG bio-plasticizers (**a**), and PLA-based plasticized composites containing MB and PEG bio-plasticizers in different ratios, CS-M and vitamin E (**b**) in the 3200–750 cm^−1^ spectral range.

The ATR-FTIR spectra of neat PLA, plasticized PLA and plasticized blends containing CS-M are given in Figure 3.

The FT-IR spectrum of the neat PLA showed a strong band at 1749 cm^−1^ due to C=O stretching of the carbonyl group, the bending vibration of this group at 1267 cm^−1^, amorphous and crystalline phases revealed at 867 cm^−1^ and 754 cm^−1^, symmetric and asymmetric CH stretching in CH_3_ at 2995 cm^−1^ and 2945 cm^−1^, and C-O stretching vibration at 1085 cm^−1^ and 1183 cm^−1^, similar with those reported in literature [55,56,57,58,59]. The introduction of MB bio-plasticizer into PLA matrix led to a decrease in the intensity of the main absorption peaks characteristic for PLA, while the presence of MB/PEG bio-plasticizers intensified this spectral behavior (Figure 3a). As the content of PEG bio-plasticizer in plasticized PLA increased, it was observed that the carbonyl band associated with the amorphous phase in PLA decreased in intensity. A more obvious effect was noted for PLA_40:60 sample, for which an increase in crystallinity was expected. Other authors did not observe different absorption peaks for plasticized PLA compared to neat PLA probably due to their ester structure similar to that of PLA [55].

The bands characteristic of the chitosan’ saccharide units appeared at 1152 cm^–1^ (anti-symmetric stretching of the C–O–C bridge), 1064 and 1023 cm^−1^ (skeletal vibration involving the C–O stretching overlapped with C–N stretching vibration) [31]. The absorption bands for PLA biocomposites containing CS-M were evidenced at 957 cm^−1^ (O-H stretching of carboxylic acid), 1041 cm^−1^, 1082 cm^−1^ and 1128 cm^−1^ (–C–O– stretching vibration in –O–C=O), 1182 cm^−1^ (the carbonyl (C-O) stretching in the carboxylic group [60]), 1362 cm^−1^ and 1456 cm^−1^ (the C-H stretching of -CH_3_, symmetric and asymmetric deformation), 1265 cm^−1^ (C-O bending), 1302 cm^−1^ (CH_3_), 1749 cm^−1^ (the stretching of carbonyl ester group (>C=O) specific both to PLA and rosehip oil [31], as well as 2995 cm^−1^ and 2945 cm^−1^ (C-H stretching of -CH_3_) [36]. The biocomposites showed the same absorption bands as PLA/CS_100:0, which are differentiated by their intensity. Compared to the plasticized PLA samples, those containing CS-M showed an increase in the intensity of carbonyl band, except for the PLA/CS-M_70:30 biocomposite.

### 3.4. Transmittance by UV/VIS Spectrometry

**Figure 4:**
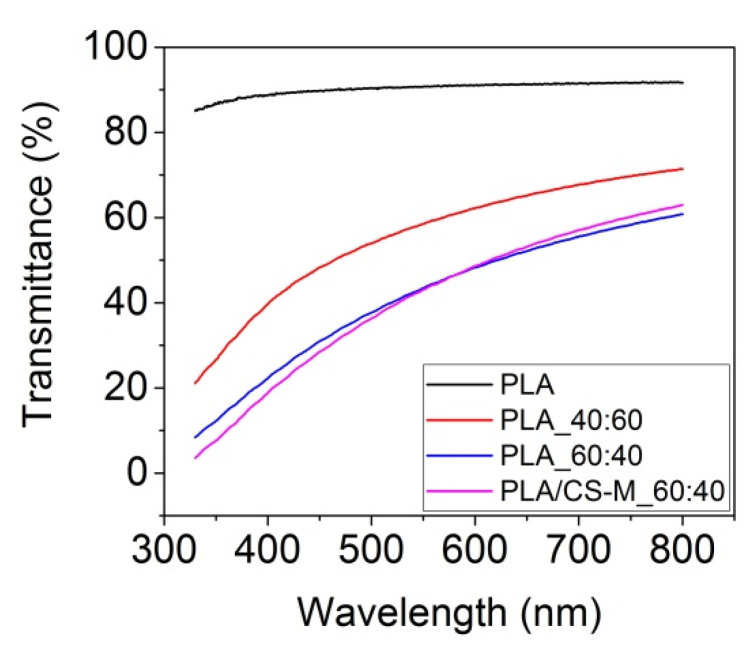
Transmittance by UV/VIS for some PLA-based materials films compared with neat PLA.

Figure 4 shows the transmittance in UV/VIS spectrometry performed for the neat PLA, PLA_60:40, PLA/CS-M_60:40, and PLA_40:60 films after Brabender processing.

Figure 4 revealed that the neat PLA film had a remarkable transmittance (~90%), while the PLA_40:60 film registered a transmittance of about 70% in visible, higher than the film containing PEG 8% (PLA_60:40). Further, the incorporation of 1 wt% CS-M into PLA matrix (PLA/CS-M_60:40) did not influence this optical characteristic. This result will be beneficial for food packaging applications when the consumers prefer transparent packaging materials.

### 3.5. Tensile Properties

**Figure 5:**
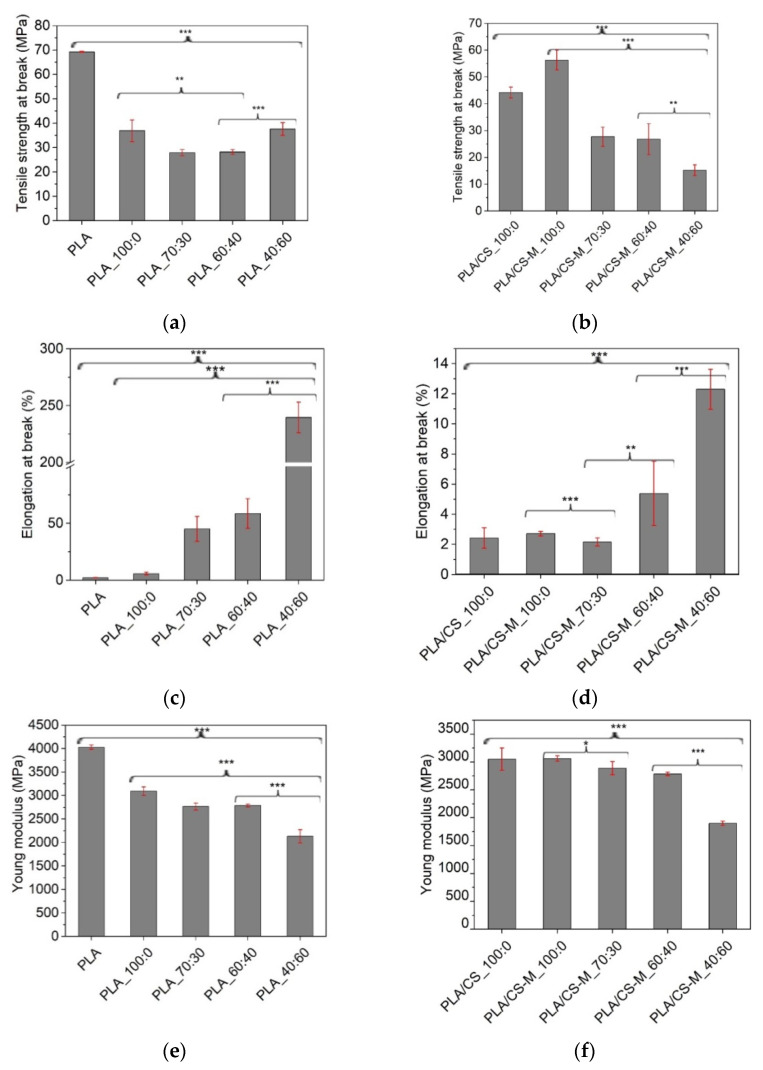
Tensile strength at break for PLA biocomposites containing MB/PEG bio-plasticizers (**a**), and same composites with CS-M (**b**), Elongation at break for PLA biocomposites containing MB/PEG bio-plasticizers (**c**), and same composites with CS-M (**d**), and Young modulus for PLA biocomposites containing MB/PEG bio-plasticizers (**e**), and same composites with CS-M (**f**). The statistical analysis was evaluated with 1-ANOVA test, where: * signifies *p* < 0.05; ** *p* < 0.01 and *** *p* < 0.001. Error bars signify the standard deviation.

The physical characteristics of polymers for packaging depend on their chemical structure, molecular weight, crystallinity, and processing conditions. Mechanical properties of biocomposites are an important factor to be studied in order to establish their potential use in the field of food packaging applications that require several characteristics for the manufacture, handling and transport of food. The most frequently evaluated properties in tensile testing are: tensile strength at break, elongation at break, and Young modulus. The influence of MB/PEG bio-plasticizers content on the tensile properties of PLA-based active biocomposites containing CS-M relative to plasticized PLA was shown in Figure 5a–f.

As a general rule, the tensile strength reduced with incorporation of bio-plasticizers [61], especially with the decrease of MB in bio-plasticizer system up to 60%. Results from Figure 5a show that the highest values of tensile strength were recorded for biocomposites containing only MB as plasticizer (36.9 ± 4.4 MPa), almost similar with the PLA_40:60 sample, i.e., 37.6 ± 2.6 MPa. As a consequence, the Young’s modulus decreased—Figure 5f, which is related to the respective change in the rigidity of the developed biocomposites by replacing a part of the brittle PLA matrix with plasticizers, vitamin E and chitosan.

It can be mentioned that, compared to the value recorded for PLA plasticized with MB, PLA_100:0, (36.9 ± 4.4 MPa), the introduction of unmodified, as well as of CS-M to the plasticized PLA had the effect of enhancing the tensile strength at break for these biocomposites, i.e., 42.2 ± 2 MPa for PLA/CS_100:0 and 56.3 ± 3.7 MPa for PLA/CS-M_100:0, respectively (*p* < 0.001) (Figure 5b).

The elongation at break for PLA_100:0 system (5.73 ± 1.14%) was slightly increased related to neat PLA (2.24 ± 0.17%) (*p* < 0.001). The increasing of PEG content in MB/PEG system significantly changed the elongation at break of plasticized PLA and PLA-based biocomposites (Figure 5c,d). A remarkable enhancement in elongation at break was observed from 5.73 ± 1.14% for PLA_100:0 sample to 239.5 ± 13.57% for PLA_40:60 sample (*p* < 0.001). The same trend was observed in the case of biocomposites containing CS-M, when the highest values of elongation at break were obtained for MB/PEG bio-plasticizers ratio with the largest amounts of PEG. The loading of 1 wt% simple chitosan in the PLA plasticized with 20 wt% MB caused a slight decrease of elongation at break to a value of 2.72 ± 0.15% for the same amount of rosehip oil encapsulated into chitosan in PLA/CS-M_100:0 biocomposite (Figure 5d). This behavior could be attributed to the interaction between chitosan filler and PLA matrix, leading to a restriction of the movement of the polymer chains. Also a small difference between mean groups was observed for the samples without or with CS-M having the ratio between MB/PEG bio-plasticizers of 70:30 compared to those with 60:40 MB/PEG ratio for all tensile properties (*p* > 0.1).

The addition of MB/PEG bio-plasticizers decreased the tensile strength and Young’s modulus (Figure 5e,f) because the plasticizers penetrated between the polymer chains and diminished the intermolecular forces that caused the cohesion of the polymer chain.

Depending on their final role, such as in this study food trays or flexible films for covering food trays, the mechanical properties of the developed biocomposites were given by the proper use of different components, as well as the bio-plasticizers ratio. By adjusting the mass ratio between MB and PEG, the elongation at break had different values, depending on the intended use of the food packaging. The PLA_40:60 sample met the requirements for use as flexible prototype film for food packaging applications, with an over 100-fold increase in elongation at break related to neat PLA. For rigid packages requirements, any formulations containing CS-M may be selected, but the final one should impart more properties besides the mechanical ones for food packaging applications. Incorporation of CS-M into plasticized PLA matrix with the MB/PEG bio-plasticizers ratio of 60:40 had a lowering effect on tensile properties. However, this formulation may be suitable for obtaining rigid packages where a tensile strength greater than 10 MPa is required.

### 3.6. DSC Measurement

**Figure 6:**
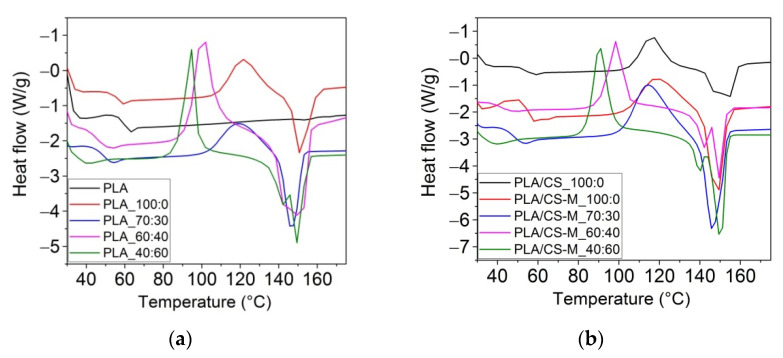
Differential scanning calorimetry (DSC) curves (exo up), the second heating run, for neat PLA, PLA with MB/PEG bio-plasticizers: (**a**), and PLA-based biocomposites containing CS-M: (**b**).

DSC scans of neat PLA, blends of PLA with bio-plasticizers and plasticized PLA containing CS-M were carried out in order to characterize the thermal properties of these developed materials. DSC curves for PLA-based active biocomposites compared to reference are illustrated in Figure 6a,b, while DSC parameters are summarized in Table 3.

As observed from Table 3, the neat PLA showed a Tg at ~60 °C due to its amorphous fraction, a small melting enthalpy and the melting temperature at ~154 °C of the crystalline PLA fraction, all values being similar with those found in other studies [62,63]. Consequently, the degree of crystallinity recorded of 0.5%, explained by the lack of recrystallization after cooling. PLA-based biocomposites melt around 146–156 °C, accompanied by a small shoulder (139–151 °C) due to melting of different types of crystallites and a cold crystallization temperature in the range of 88–118 °C. Also, analyzing the DSC data one can observe that the addition of PEG bio-plasticizer led to a decrease of glass transition temperature, for example for PLA_70:30 (49.4 °C), PLA_60:40 (46.8 °C), and PLA 40:60 samples (48.1 °C) compared to neat PLA. Incorporation of CS-M caused the restriction of macromolecular chains mobility and the melting peaks in the same range as plasticized sample. It is also found that the introduction of CS-M led to the decrease of the glass transition temperature compared to the sample containing simple chitosan, reducing also the crystallinity of PLA. The highest degree of crystallization, associated with the highest amount of PEG was obtained for PLA_40:60 sample (5.0%). This was correlated with the decrease in the intensity of the amorphous carbonyl (1749 cm^−1^). A high degree of crystallinity is reflecting in the improvement of the barrier properties of the films because the water diffuses mainly through the amorphous regions (the crystals are considered to be impermeable). The PLA/CS-M_60:40 and PLA_40:60 formulations gave the Xc values of 3.9% and 5.0%, respectively.

The Tcc for the plasticized PLA samples having dual bio-plasticizers with PEG content in large amounts, i.e., PLA_40:60, PLA_60:40, as well as those containing CS-M decreased compared to the same thermal parameter recorded for PLA plasticized only with MB (PLA_100:0). This behavior was due to the increase in the PLA chain mobility due to the PEG bio-plasticizer, which certified the crystallization of PLA due to the presence of short chains [63]. The effect of Lapol^®^108 on the decrease the T_CC_ of plasticized PLA-PHB blends was also observed by Abdelwahab et al. [61].

### 3.7. WVTR

The water vapor barrier property of the films is important for products whose physical and chemical damage is caused by the moisture content at equilibrium, as well as for materials subjected to moisture conditions during storage and/or transport. Table 4 summarizes the data of water vapor permeability for neat PLA and plasticized PLA-based biocomposites, which reveal the possibility of balance the permeability by acting on the composition.

The introduction of the CS-M into the plasticized PLA matrix has generally led to slight increased water vapor permeability with respect to the corresponding plasticized samples, which is due to the high affinity of chitosan for water [33]. PLA/CS-M_100:0 showed the enhanced water vapor barrier, i.e., a WVTR value of 17.10 g m^−2^ day^−1^ compared to the same material containing unmodified chitosan, i.e., 25.31 g m^−2^ day^−1^. The ANOVA statistical analysis showed that there is a significant difference (*p*-value < 0.001) between the WVTR of samples.

The water vapor barrier has a great impact for maintaining or extending the shelf-life of the packaged food product, influencing the physical and chemical deteriorations by equilibrium moisture content [64]. The water presence in semi-crystalline polymers such as PLA mainly affects the amorphous region, breaking-up the hydrogen bonds between the polymer chains, thus resulting in a reduction in the barrier properties of the material [65]. Crystal polymorphism (α and α’ form) influenced the barrier properties and mechanical performance of poly(L-lactic acid) as found by Cocca et al. [66]. The water vapor permeability is affected by the polymorphic structure, the diffusion of the adsorbed water vapor reducing due to tighter molecular packing chain segments, along with strong coupling with the amorphous parts of the macromolecules.

The results showed that by increasing the PEG bio-plasticizer content in PLA biocomposites, the water vapor permeability increased, from 15.94 g m^−2^ day^−1^ for the neat PLA to 21.97 g m^−2^ day^−1^ in the case of 70/30 ratio between plasticizers, to 28.09 g m^−2^ day^−1^ when the ratio between plasticizers is 60/40 and to 37.45 g m^−2^ day^−1^ respectively if the plasticizers ratio was 40/60. This result is consistent with literature data which showed an increase in WVTR with the plasticization of PLA [67]. This increase in water vapor permeability was correlated with the decrease in the degree of crystallinity reported in Table 3.

Permeability (P) depends directly on the solubility (S) and diffusion coefficients (D) of permeating molecule (P = D × S), D being influenced by the polymer structure, including free volume and tortuosity, while solubility coefficient depends on the permeant’s solubility at molecular scale [68]. Knowing that vitamin E and rosehip oil are not water soluble, one can say that the rise of WVTR was not given by the solubility, but the diffusivity of water vapor molecules through the plasticized PLA chains. The used MB and PEG bio-plasticizers extended the PLA polymer chains mobility, as well as free volume, the diffusion of water molecules being facilitated through the interstitial space. Therefore, the highest value of water vapor permeability was reached for an increased content of PEG (40:60), with more pathways being available for water diffusion in PLA_40:60 and PLA/CS-M_40:60 composites [69].

### 3.8. Radical Scavenging Activity (RSA (%)) Assay

The antioxidant efficiency of the PLA-based composites was determined based on the reaction with 2,2-diphenyl-1-picrylhydrazyl (DPPH˙) free radical.

The results from Table 5 show that the antioxidant activity decreased with the lowering of PEG content from MB/PEG system. DPPH test evidenced the effective radical scavenging activity of the samples containing bioactive components in composition.

The results of UV–Vis spectroscopy investigation showed that DPPH radicals were partially quenched and the differences between samples were not very significant, probably due to the similar content of vitamin E of plasticized PLA and composites with CS-M, as well as due to incorporation of a small amount (1%) of chitosan encapsulated with rosehip oil.

The antioxidant feature of the developed biocomposites was imparted by a combined effect resulted by simultaneous use of rosehip oil encapsulated into chitosan and vitamin E. However, the effect of introduction CS-M into PLA was remarkable compared with sample containing unmodified chitosan. Improving the antiradical effect by adding chitosan in PLA-based formulations, along with enhancement antioxidant activity due to synergistic interactions of chitosan with rosemary ethanolic extract was also reported in our previous study [70]. Blends with rosehip seed oil encapsulated into chitosan mostly exhibited a higher level of radical scavenging activity compared to the same samples without modified chitosan. The biocomposite containing the MB/PEG bio-plasticizers ratio of 40:60 and modified chitosan had a higher antioxidant activity (28.92%). The antioxidant and antimicrobial activities of chitosan were linked to the presence of amino and hydroxyl groups in the chitosan structure [71]. The use of phenolic type natural antioxidants from plant species, in our case rosehip oil, is beneficial for producing food packaging materials. Vitamin E is a chain-breaking antioxidant that acts as a free radical scavenger, inhibiting the production of reactive oxygen species molecules during the propagation of free radical reactions and protecting against polyunsaturated fatty acids peroxidation in cell membranes [72]. The sample containing CS-M and MB/PEG bio-plasticizers ratio of 60:40 was distinguished by a more pronounced antioxidant activity (36.27%). It is also observed from the data of the Table 5 that the antioxidant activity decreased with an increase of the PEG content from MB/PEG bio-plasticizers, i.e., from 29.41% in the case of PLA/CS-M_100:0 biocomposite to 25.98% for PLA_40:60 sample.

### 3.9. Antimicrobial Activity

Food spoilage can also be caused by microbial growth on the surface of food. Packaging films with antimicrobial properties can prevent such damage. The results of the antibacterial tests of PLA-based materials performed according to ISO 22196:2007 are presented as log reduction of the *Escherichia coli* ATCC 8739 and *Staphylococcus aureus* ATCC 6538 (Table 6).

Samples containing cold-pressed rosehip oil encapsulated into chitosan showed a significant antimicrobial activity against *S. aureus* (logarithmic reduction of 2.6–3.1). The antimicrobial activity felt within the range of logarithmic reduction of 1.3–2.8 compared to *E. coli* and 2.5–3.1, respectively, compared to *S. aureus.* Log reductions of 2.73 and 5.54 against *S. aureus* and *E. coli* respectively were found for 1 wt% unmodified chitosan incorporated into PLA/tributyl *o*-acetyl citrate (ATBC) biocomposite by melt mixing technique [36]. Higher antibacterial activity was also reported in the case of the essential and vegetable oils (clove and argan oils) encapsulated into chitosan and deposited onto PLA film by the electrospinning technique [73].

### 3.10. Development of Food Tray and Film

Among the physical properties, the packaging materials must demonstrate good processing on the existing machines (low viscosity in the melt) and satisfactory hardness and flexibility as to guarantee their handling. Further, they must ensure the sealability of the packaging. In relation to the specific requirements for final applications as food tray or flexible film, based on the overall properties, but mainly on the physical-mechanical test results, PLA_CS-M_60:40 and PLA_40:60 samples were selected as the best formulations to obtain prototypes for food trays by thermoforming technology and film by pressing, respectively. The optimization of the technological parameters consisted in the monitoring and regulation of the melting temperature on the eight areas of the screws, of the feeding speed, of the mixing speed and of the pressure. Table 7 shows the optimal technological parameters for obtaining the granules from the two selected recipes.

The food trays with dimensions of 140 × 70 × 40 mm and uniform thickness of ~0.5 mm were obtained on the thermoforming machine, by using) a temperature of 110 °C, followed by trimming with laser. Flexible and transparent films made of PLA_40:60 formulation with dimension of 300 × 300 × 0.1 mm have been produced by pressing.

**Figure 7:**
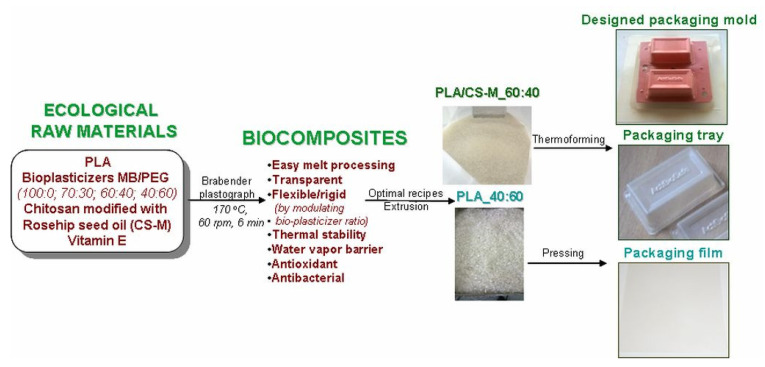
Overview of the main technological steps for obtaining of food packaging system.

Figure 7 shows the main materials obtained by compounding and pressing technologies for obtaining food tray and film for covering.

The successful produced food trays and covering films containing the optimum developed formulations presented in this study will be further tested for food packaging applications focusing on safety and increased shelf-life (physical-chemical, sensory, and microbiological analyses used to determine shelf-life of food, migration of components into food) and validation for industrial scaling (vacuum packaging, sealing tests, thermal and freezing stability).

## 4. Conclusions

This study was carried out in order to protect the environment and consumer safety, based on the following particular characteristics:

(i) The raw materials used (PLA, MB, and PEG bio-plasticizers, chitosan, cold-pressed rosehip seed oil, vitamin E) were derived from non-toxic renewable resources.

(ii) The method of obtaining the polymer blends by melt processing was relatively simple, without any toxic solvent use.

(iii) By adjusting the ratio of MB and PEG bio-plasticizers in recipes, a modulation of flexible or rigid food packaging could be achieved, e.g., the 40/60 ratio between MB and PEG bio-plasticizers has led to the production of flexible films, while the 60/40 ratio between MB and PEG bio-plasticizers, together with the incorporation of CS-M was found to be suitable for tray production.

(iv) Chitosan itself is a bioactive antibacterial component and, by encapsulating of rosehip seed oil derived from cold-pressed seeds into it, acted synergistically to create a bioactive food packaging.

(v) Small amounts of antimicrobial/antioxidants agents have been used to reduce the number of microorganisms on the surface of food packaging and implicitly further on packaged food product with consequences for increasing the shelf life of the packaged product and on the health and safety of consumers.

The obtained biocomposites based on PLA, CS-M, and additives showed the improved processability and physical-mechanical, thermal, water vapor barrier, antioxidant, and antimicrobial activities suitable for use in food packaging.

Studies have led to the development of optimized processing conditions and the most appropriate formulations based on plasticized PLA and antimicrobial/antioxidant agents to obtain food tray prototypes (PLA/CS-M_60:40) and flexible films (PLA_40:60) by thermoforming and pressing technologies.

## 5. Patents

Rapa, M., Vasile, C., Grosu, E., Trifoi, A.R., Darie-Nita, R.N., Butnaru, E., Dumitriu, R.P., Sivertsvik, M., Rosnes, J.T., Mitelut, A.C., Popa, E.E., Popa, M.E., Munteanu, B., Moldovan, L. PLA—based active and degradable biocomposites for food packaging. Patent WO/2018/117885/28.06.2018, international application no. PCT/RO2016/000028/27.12.2016.

## Figures and Tables

**Table 1:** Compositions of developed PLA-based materials.

Sample	PLA (wt%)	MB (wt%)	PEG (wt%)	CS or CS-M (wt%)	Vitamin E (wt%)
PLA	100	-	-	-	-
PLA_100:0	80	20	-	-	-
PLA/CS_100:0	78.4	19.6	-	1	1
PLA/CS-M_100:0	78.4	19.6	-	1	1
PLA_70:30	79.2	13.86	5.94	-	1
PLA/CS-M_70:30	78.4	13.72	5.88	1	1
PLA_60:40	79.2	11.88	7.92	-	1
PLA/CS-M_60:40	78.4	11.76	7.84	1	1
PLA_40:60	79.2	7.92	11.88	-	1
PLA/CS-M_40:60	78.4	7.84	11.76	1	1

**Table 2:** Melt processing characteristics of PLA-based materials containing MB and PEG bio-plasticizers, in different ratios, CS-M and vitamin E.

Sample	TQ_max_ (N m)	TQ_end_ (N m)	Melt Viscosity, η (N m rpm^−1^)	Power, P (kW)
PLA	52	34	0.56	0.213
PLA_100:0	60	31	0.51	0.194
PLA/CS_100:0	55	33	0.55	0.207
PLA/CS-M_100:0	45	31	0.51	0.194
PLA_70:30	39	30	0.50	0.188
PLA/CS-M_70:30	65	32	0.53	0.200
PLA_60:40	53	28	0.46	0.175
PLA/CS-M_60:40	52	30	0.50	0.188
PLA_40:60	46	27	0.45	0.169
PLA/CS-M_40:60	40	28	0.46	0.175

**Table 3:** Thermal parameters evaluated from DSC curves for PLA-based biocomposites plasticized PLA and neat PLA.

Sample	Tg (°C)	ΔHcc (J/g)	Tcc (°C)	ΔHm (J/g)	Tm (°C)	Xc,_PLA_ (%)
PLA	60.7	-	-	0.52	154.1	0.5
PLA_100:0	52.9	32.5	121.3	34.7	151.0; 157.0	2.9
PLA/CS_100:0	53.4	28.7	116.0	29.3	156.2; 148.9	0.8
PLA/CS-M_100:0	55.0	16.4	118.4	16.7	148.2	0.4
PLA_70:30	49.4	14.5	119.2	17.5	146.6	4.0
PLA/CS-M_70:30	48.2	21.7	114.3	23.9	146.2	3.0
PLA_60:40	46.8	24.0	100.3	26.5	151.4; 143.6	3.3
PLA/CS-M_60:40	51.3	20.5	106.1	23.4	146.0; 151.8	3.9
PLA_40:60	48.1	28.4	90.3	29.3	150.3; 139.2	1.2
PLA/CS-M_40:60	43.6	22.1	88.2	25.8	150.3; 139.2	5.0

**Table 4:** Water vapor permeability of PLA-based biocomposites, plasticized PLA and neat PLA.

Sample	WVTR (g m^−2^ Day^−1^)
PLA	15.94
PLA_100:0	30.86
PLA/CS_100:0	25.31
PLA/CS-M_100:0	17.10
PLA_70:30	21.97
PLA/CS-M_70:30	27.59
PLA_60:40	28.09
PLA/CS-M_60:40	31.21
PLA_40:60	37.45
PLA/CS-M_40:60	38.95

**Table 5:** Radical scavenging activity (RSA (%)) for PLA-based biocomposites and plasticized PLA samples. Reported values correspond to means (±standard deviation).

Sample	RSA (%)
PLA/CS_100:0	15.49 ± 0.005
PLA/CS-M_100:0	29.41 ± 0.009
PLA_70:30	27.45 ± 0.012
PLA/CS-M_70:30	25.98 ± 0.017
PLA_60:40	26.35 ± 0.053
PLA/CS-M_60:40	36.27 ± 0.053
PLA_40:60	25.98 ± 0.025
PLA/CS-M_40:60	28.92 ± 0.028

**Table 6:** Log_10_ reduction of *Escherichia coli* ATCC 8739 and *Staphylococcus aureus* ATCC 6538 in contact tests with PLA-based biocomposites.

Sample	Log_10_ Reduction of the Number of Viable Bacterial Cells	
	*E. coli* ATCC 8739	*S. aureus* ATCC 6538
PLA/CS-M_100:0	2.42 (>99%)	-
PLA_70:30	1.30 (>90%)	2.50 (>99%)
PLA/CS-M_70:30	1.80 (>90%)	3.00 (>99.9%)
PLA_60:40	1.67 (>90%)	-
PLA/CS-M_60:40	0.57 (<90%)	-
PLA_40:60	2.80 (>99%)	2.60 (>99%)
PLA/CS-M_40:60	2.20 (>99%)	3.10 (>99.9%)

**Table 7:** Technological parameters for compounding of PLA/CS/M_60:40 and PLA_40:60 formulations.

Technological Parameters	Values	
	Film (PLA_40:60)	Trays (PLA/CS-M_60:40)
Temperatures on zones (°C)	113/137/163/162/168/168/167/163	58/89/159/160/166/167/164/141
Temperature at die (°C)	131	147
Pressure at die (MPa)	13.85	12.6
Engine speed (rpm)	270	315
Screw speed (rpm)	67.5	78

## Data Availability

The data presented in this study are available on request from the corresponding author.
